# How collaborative mental health care for competitive and high-performance athletes is implemented: A novel interdisciplinary case study

**DOI:** 10.3389/fpsyg.2022.994430

**Published:** 2022-12-20

**Authors:** Krista J. Van Slingerland, Poppy DesClouds, Natalie Durand-Bush, Véronique Boudreault, Anna Abraham

**Affiliations:** ^1^SEWP Lab, School of Human Kinetics, Faculty of Health Sciences, University of Ottawa, Ottawa, Ontario, Canada; ^2^Faculty of Physical Activity Sciences, Université de Sherbrooke, Quebec, Canada; ^3^Department of Sports Services, University of Ottawa, Ottawa, Ontario, Canada

**Keywords:** mental health, mental illness, sport psychology, anxiety, treatment, case study

## Abstract

**Introduction:**

Collaborative care is considered a best practice in mental health care delivery and has recently been applied in high-performance sport to address athletes’ mental health needs. However, how the collaborative process unfolds in practice in the sport setting has not yet been well documented. The purpose of this illustrative case study was to investigate a novel interdisciplinary approach used within the Canadian Centre for Mental Health and Sport (CCMHS) to provide mental health care to clients. Focusing on ‘how’ the approach was implemented, the aim of the study was to provide insight into the collaboration that occurred between mental performance and mental health practitioners to provide care to a high-performance athlete over an 11-month period, as well as factors facilitating and impeding the team’s collaboration. The case involved three practitioners and a 16-year-old female athlete experiencing chronic pain, low mood, and elevated anxiety.

**Methods:**

In the first phase of the data collection process, each practitioner engaged in guided reflective journaling to describe the case and reflect on their practice and outcomes. During the second phase, practitioners co-created a case timeline to describe the collaborative process using clinical documents. Lastly, practitioners participated in collaborative reflection to collectively reflect more broadly on collaboration practice occurring within the CCMHS and Canadian sport system.

**Results:**

The data depict a complex care process in which the necessity and intensity of collaboration was primarily driven by the client’s symptoms and needs. A content analysis showed that collaboration was facilitated by the CCMHS’ secure online platform and tools, as well as individual practitioner and team characteristics. Collaboration was, however, hindered by logistical challenges, overlapping scopes of practice, and client characteristics.

**Discussion:**

Overall, there were more perceived benefits than drawbacks to providing collaborative care. While flexibility was required during the process, deliberate and systematic planning helped to ensure success. Factors such as interdependence of collaborative practice, complementarity of practice within care teams, compensation for collaboration, in-person versus virtual delivery, and intricacies of care coordination should be further examined in the future to optimize collaborative mental health care in sport.

## Introduction

### Collaborative care

Collaborative approaches to care have emerged globally as a best practice (Bullock et al., 2017), particularly when it comes to the provision of mental health care in conjunction with primary care (e.g., Thota et al., 2012). Collaborative practice is an interprofessional process of communication and decision-making that integrates the separate and shared knowledge and skills of care providers to inform client care (Way et al., 2000). Collaborative practice, as a dynamic process, occurs on a spectrum ranging from independent parallel practice to consultation, referral, and interdependent co-provision of care (Lorenz et al., 1999; Way et al., 2001). *Independent parallel practice* refers to care provided by a single practitioner acting within their professional scope of practice, which is sufficient to address a client’s challenges. When the skill set of one practitioner is insufficient or additional support is desired to address the complexities of a client’s needs, health care providers may *consult with* or *refer* the client to another professional whose unique competencies are required. *Co-provision of care* represents the highest degree of interdependence and is characterized by shared decision-making, respect for the unique contributions of all practitioners and bi-directional consultation and referral. When care is shared in this way, clients are familiar with all providers and often choose which practitioner they engage with based on their needs and provider availability Jones and Way, (2006).

Collaborative care models are also used in sport, where it is common for sports medicine physicians, surgeons, athletic therapists, strength and conditioning experts and coaches to work together to optimize athlete performance and recovery (Reid et al., 2004). More recently, collaborative care models have been applied to support athletes experiencing mental health^1^ and mental performance^2^ challenges, as well as symptoms of mental illness^3^ (Moesch et al., 2018; Van Slingerland and Durand-Bush, 2021). Interprofessional teams that include experts from different domains (e.g., mental performance and mental health) who are able to address the dual aim of optimizing athletic performance and overall wellness are becoming increasingly common in high-performance sport (Dijkstra et al., 2014). There is a lack of consensus, however, as to whether mental performance and health mental services ought to be offered separately but in a complementary fashion by different practitioners, or integrated and offered by a single practitioner with dual expertise (McHenry et al., 2021). This uncertainty has sometimes resulted in a tension felt by mental performance and mental health practitioners working in the field of sport and by sport organizations who lack clear, evidence-based direction to support decision-making. Consequently, additional data that shed light on the benefits and drawbacks of different models of mental performance and mental health service provision are needed to inform holistic service development and delivery in high-performance sport settings.

### The CCMHS collaborative care model

Collaborative care is a central feature of the model used at the CCMHS⎯ a registered charity dedicated to the provision of mental health care and resources within the Canadian sport system (Durand-Bush and Van Slingerland, 2021a). This national center was created based on a Participatory Action Research (PAR) project carried out with several stakeholders working in the areas of sport and mental health (Van Slingerland et al., 2021). The research project focused on the design, implementation, and evaluation of a collaborative sport-centered mental health care model for competitive and high-performance athletes (Van Slingerland and Durand-Bush, 2021). In line with best practices, the model is interdisciplinary, person-centered, and includes a variety of practitioners capable of addressing mental health, mental illness, and mental performance [e.g., psychotherapists, counsellors, psychologists, psychiatrists, mental performance consultants (MPCs)]. There is also a multidisciplinary element to the care model allowing the core mental health team of practitioners to collaborate with a client’s extended mental health care team (e.g., physicians, dieticians, physical therapists) and support network (e.g., coaches, family members) as necessary if the client wishes and consents to this.

The CCMHS collaborative care model is based on the following key characteristics, which were established in the PAR research project (Van Slingerland et al., 2021; Van Slingerland and Durand-Bush, 2021): sport-centered care, collaborative care, and in-person and virtual nationwide care.

#### Sport-centered care

Services provided through the CCMHS are sport-centered because studies have revealed that athletes prefer and benefit from working with practitioners who understand competitive sport (Gavrilova and Donohue, 2018; Moesch et al., 2018; Jewett et al., 2020; Van Slingerland et al., 2020). According to Jewett et al. (2020), high-performance athletes reported a need to be supported by mental health practitioners who understand their world. This allows practitioners to be efficient and adapt their therapeutic approaches to meet evolving sport-specific issues and demands.

#### Collaborative care

Collaboration is another key characteristic of services provided through the CCMHS. Practitioners rely on their knowledge and skills to work in a complementary fashion with other team members to optimize care processes and outcomes (Nancarrow et al., 2013; Van Slingerland and Durand-Bush, 2021). Importantly, practitioners within each care team have the autonomy to determine the amount of collaboration deemed necessary to deliver a comprehensive mental health care plan (Way et al., 2000). The plan takes into account “the complex roles, identities, and demands that athletes or coaches must manage within their sport system and culture throughout a competitive season and quadrennium” (Durand-Bush and Van Slingerland, 2021a, p. 87). The care team is managed by the CCMHS Care Coordinator who conducts intakes with clients, assigns clients to a care team, communicates with clients and practitioners as necessary throughout the care process to meet their evolving needs, and monitors data to foster effective and efficient evidence-based practice (Van Slingerland et al., 2020; Durand-Bush and Van Slingerland, 2021a).

#### In-person and virtual Nationwide care

Another important feature of the CCMHS model is the provision of care across Canada’s different provinces and territories both in person and virtually through a secure telehealth platform. This is essential given the national mandate of the CCMHS, the frequent relocation of athletes and coaches to train and compete, and the interjurisdictional restrictions by which psychologists must abide. The systematic but flexible process followed by the care team ensures that clients can quickly access their team of practitioners wherever they are, and the team can also be adapted if necessary to meet changing needs and challenges (Van Slingerland and Durand-Bush, 2021). The typical timeline between client referral and the onset of care is two weeks, which is exceptional compared to current wait times for mental health services in Canada. While access to services varies based on geographical area, average wait times for children and youth are 67 days for counselling and therapy, and 92 days for intensive treatment (Center for Addiction and Mental Health, n.d.).

#### Client mental health care pathway

**Figure 1:**
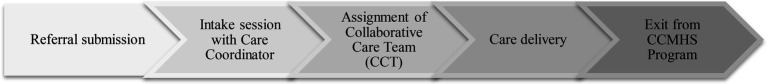
CCMHS mental health care pathway.

Figure 1 provides a global view of the CCMHS care pathway from client referral to exit. In a previous case study conducted by Van Slingerland et al. (2020), the authors demonstrated how prospective clients can self-refer or be referred by an ally (e.g., coach, parent, MPC) either online, by phone, or email. Prospective clients are then invited to complete an intake interview with the Care Coordinator as well as an online survey to assess their mental health, mental performance, and mental illness symptoms (e.g., anxiety, depression, eating behaviours). The Care Coordinator then uses this information to assign the client to a Collaborative Care Team (CCT) that includes a minimum of two practitioners (a lead and support). Care is then delivered by the CCT until the client exits the CCMHS program, although the client can return at any point if additional support is required. A robust explanation of the intake process, including the eligibility criteria that must be met by prospective clients is outlined in the aforementioned case study by Van Slingerland et al. (2020) as well as in Van Slingerland and Durand-Bush’s (2021) study on the design of the CCMHS and collaborative care model.

#### Evidence-based care

Importantly, the CCMHS collaborative care model was empirically evaluated (Van Slingerland and Durand-Bush, 2021). Based on findings from this evaluation, the authors concluded:

This model was the first of its kind to be systematically designed, implemented and evaluated to provide care to athletes experiencing mental health challenges and disorders. Overall, findings show that the model was acceptable and appropriate and features of the model (i.e., collaborative, sport-centered, nationwide, virtual and in-person care) should be maintained. Nonetheless, some aspects of the model can be improved, including remuneration for collaboration, subsidization of care for service-users, and efficiency of processes (Van Slingerland and Durand-Bush, 2021, p. 14).

The implementation of the model is ongoing and the CCMHS team has been adapting processes along the way to improve the efficiency of care and attenuate any unnecessary burden placed on clients and practitioners. However, it is important to note that the literature lacks empirical and practical information guiding the development and implementation of collaborative mental health care involving both mental performance and mental health practitioners working with athletes. This was the impetus to carry out the current illustrative case study.

### Purpose of study

In line with the above-mentioned gaps in the literature, this study aimed to examine the care provided to an athlete by a collaborative mental health care team comprised of CCMHS mental performance and mental health practitioners, and identify factors that facilitated and impeded collaboration.

## Methodology

An illustrative case study design was used to describe the processes, events, and outcomes associated with the provision of care over an 11-month period (Harrison et al., 2017). This type of case study allows researchers to holistically illustrate the evolution of complex issues and experiences in real-life settings (Stake, 2006). The current case was selected to demonstrate the complexity and fluctuations in client challenges and symptoms, which necessitated a high degree of collaboration amongst the three practitioners assigned to this athlete’s care team. The collaborative practice between the mental performance and mental health practitioners was also contextualized within broader service-provision in the Canadian context.

### Case study team

The Case Study Team (*N* = 5) was comprised of two researchers and three practitioners (i.e., a MPC, a psychotherapist with the dual credential of MPC, and a clinical psychologist with the dual credential of MPC). The three CCMHS practitioners also formed the athlete’s Collaborative Care Team (CCT). The roles and characteristics of each member of the CCT are explained in a subsequent section. The two researchers have research expertise in the areas of mental health and mental performance in sport. The lead researcher (first author) is the Mental Health Manager for Game Plan, a national program responsible for national team athletes’ total wellness. The other researcher (third author) is a senior sport psychology professor at the University of Ottawa who has also been working as a MPC for 27 years. As the co-founders of the CCMHS, these two individuals provided valuable perspectives on the research process and collaborative practice between mental performance and mental health practitioners within the CCMHS and broader Canadian sport system. The lead researcher also provided administrative support to the CCT. The Case Study Team members have previous experience conducting case study research (e.g., Van Slingerland et al., 2020).

### Data collection and analysis

**Figure 2:**
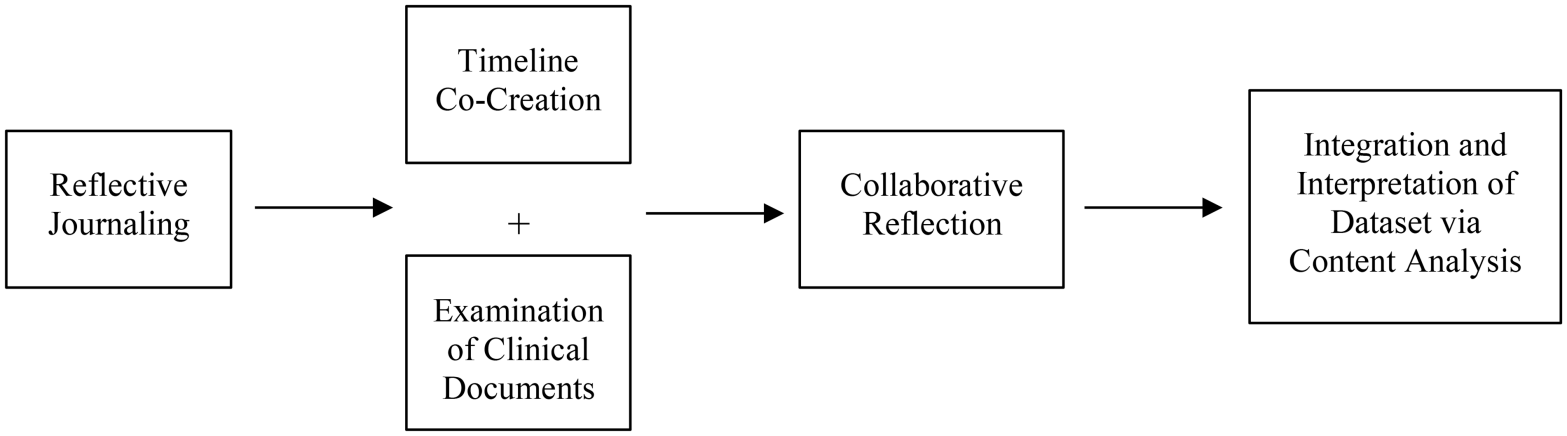
Data collection and analysis.

When conducting case studies, researchers typically adopt a pragmatic approach and employ multiple methods to gather a plethora of perspectives that provide an in-depth understanding of the phenomenon of interest (Thomas, 2011). Multiple data sources ensure that the topic is comprehensively explored, enhancing trustworthiness and allowing for various facets of the phenomenon to be revealed and understood (Baxter and Jack, 2008). The present case study was conceptualized through a multi-stage, multi-methods process of data collection and analysis (Figure 2), which were guided by a pragmatic approach and are described next.

#### Reflective journaling

At the onset of this case study, the three CCT practitioners engaged in guided reflective journaling (Yinger and Clark, 1981), a methodological approach that provides practitioners an opportunity to critically examine and make meaning of their applied experiences, with the aim of supporting their learning and future practice. The practitioners answered seven open-ended questions designed to promote critical reflection and address the objectives of the case study. The questions prompted practitioners to describe (a) the case in their own words, (b) the process of collaborative care that took place, (c) the outcomes of collaboration, and (d) the factors that impeded and facilitated collaborative practice. This exercise yielded 30 pages of double-spaced text, averaging 1,112 words per document.

#### Timeline co-creation

Next, the first author met with the CCT to collaboratively construct a timeline of the case, including the pacing and focus of care sessions, the trajectory and fluctuations in the client’s symptoms, and instances of collaboration between practitioners (i.e., when, why, and how). This provided insight into the CCT’s collective decision-making process. The timeline constructed was further informed by the examination of 28 clinical documents (Bowen, 2009) such as session notes (summaries of client care sessions), encounter notes (summaries of client encounters outside of regularly scheduled sessions), team consult notes (summaries of consultations between CCT members or between CCT members and outside healthcare providers), and other clinical records (e.g., medication) contained in the client’s Electronic Health Record (EHR).

#### Collaborative reflection

Lastly, practitioners, together with the first author, engaged in a group discussion (Bloor et al., 2001; Collin and Karsenti, 2011) to reflect more broadly on collaborative practice within the context of the CCMHS and the Canadian sport context. Five questions guided the dialogue, prompting the group to discuss their profession’s stance on collaborating with other health care professionals, their collaborative practice experience within the CCMHS, and factors they perceived to facilitate and hinder the collaborative process (e.g., CCMHS collaborative care model, EHR system, Canadian healthcare system boundaries). The discussion lasted 30 min and was recorded and transcribed verbatim.

#### Integration and analysis

The data collected *via* reflective journaling and collaborative reflection were integrated by the first author and examined in a chronological fashion to confirm the co-constructed timeline and the collaboration that occurred throughout this case. The data were also analyzed using conventional content analysis to extract codes and identify themes to address the study aims (Hsieh and Shannon, 2005). To this end, the first author immersed herself in the data, developed codes, which she then grouped into common themes addressing the CCT’s provision of collaborative care and factors influencing it. The rest of the Case Study Team acted as “critical friends” (Smith and McGannon, 2018), reviewing the codes, and discussing and refining the suggested themes over several iterations until a consensus was reached that the presentation of data was accurate and coherent.

## The case

This case examines 11 months of collaborative care provided to the client beginning in April 2019 when she entered the CCMHS system and ending in March 2020 when practitioners completed the collaborative reflection exercise, thus ending the data collection phase of the study.

### The athlete

The athlete receiving care through the CCMHS was a 16 year-old female who competed nationally in her sport and spent approximately 42 h per week training and competing in her sport.^4^ She experienced persistent pain and was supported by her parents, a family physician who managed her medication, and an MPC.

### Presenting concerns

The athlete was referred to the CCMHS by her MPC who noticed she was experiencing symptoms of depression and anxiety that were impairing her functioning in sport and life. The athlete’s scores from the CCMHS intake survey revealed symptoms of anxiety, burnout, and depression which warranted follow-up (see Table 1). The athlete also disclosed a history of self-harm and suicidal ideation, though she was stable at intake. After the onset of care, the client’s CCT noted other concerns such as challenges building trusting relationships, returning to sport following injury, managing changing life events, and coping with negative thoughts and emotions.

**Table 1:** Results of client’s intake survey.

Screening tool	Symptom	Score	Symptom level	Possible range
GAD-7	Anxiety	10	Moderate	0–21
ABQ	Burnout	3.2	Moderate	1–5
PHQ-9	Depression	12	Moderate	0–27

Results only includes scores from the intake survey for which client screened positive; GAD-7 = Generalized Anxiety Disorder scale (Spitzer et al., 2006); ABQ = Athlete Burnout Questionnaire (Raedeke and Smith, 2001); Patient Health Questionnaire (Spitzer et al., 1999).

### The CCT

Table 2 summarizes each of the three practitioner’s characteristics and the number of sessions in which they contributed to care. Practitioner A is a professional member of the Canadian Sport Psychology Association (CSPA) and the referring MPC in this case. She had been working with the client in a team setting for a year prior to noticing signs that the client required clinical mental health support. With the client’s consent, Practitioner A referred her to the CCMHS so she could benefit from working with a collaborative care team comprised of both mental performance and mental health practitioners. Since Practitioner A worked at the CCMHS in addition to having her own private practice, she was able to seamlessly act as a support practitioner on the client’s CCT. Given her scope of practice, Practitioner A focused mainly on mental performance training and sport-specific concerns during her sessions with the client. Practitioner B is also a MPC registered with the CSPA and a licensed psychotherapist working for the CCMHS and with university student-athletes. When the CCT was established by the CCMHS Care Coordinator, Practitioner B was assigned as the lead practitioner. Practitioner C is a clinical psychologist in private practice with the dual credential of registered MPC who equally works for the CCMHS. At the onset of care, she was a support practitioner on the client’s CCT.

**Table 2:** Summary of practitioner characteristics and case involvement.

Practitioner	Designation	Client sessions
A	MPC	6
B	Psychotherapist, MPC	4
C	Clinical psychologist, MPC	7

### Case timeline and collaborative care

**Figure 3:**
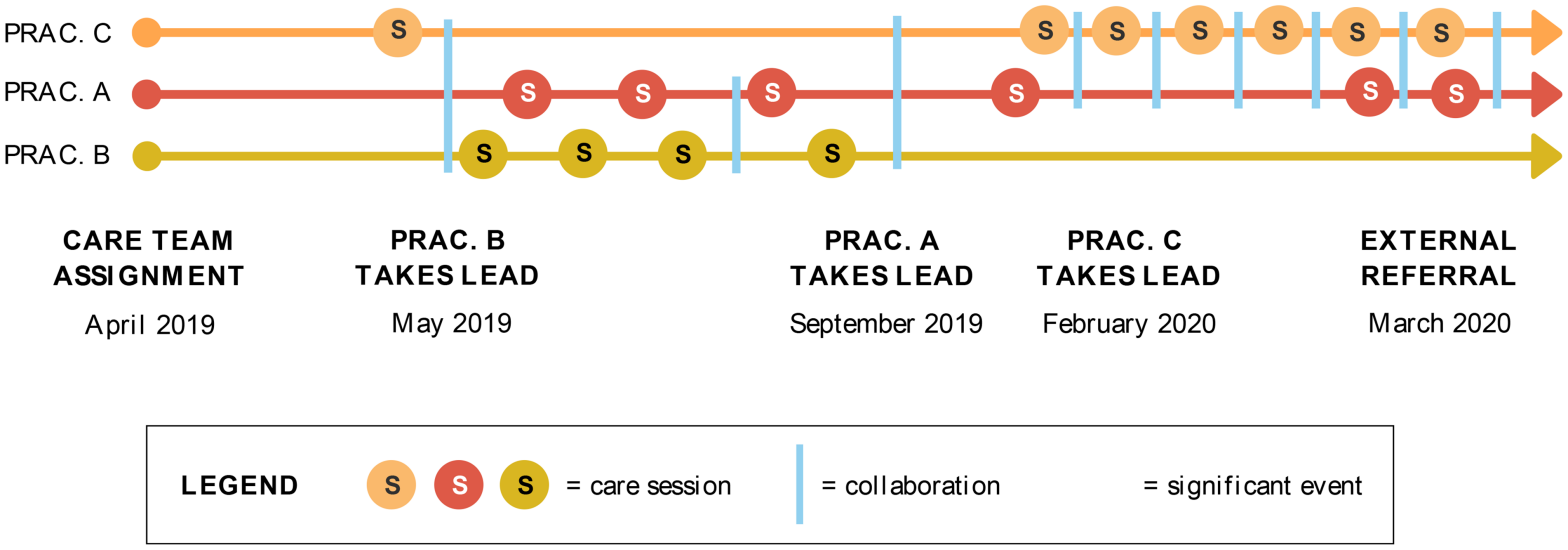
Case timeline.

Figure 3 depicts the case timeline and practitioners’ involvement within the CCT. Overall, the CCT provided 17 care sessions (represented by circles with an “s”) and collaborated nine times (represented by blue bars); the client experienced varying symptoms of depression, anxiety, and burnout and four “significant events” (e.g., disclosure of suicidal thoughts, represented by comet symbols). All significant events are plotted on Practitioner A’s timeline because the client chose to disclose distress to this practitioner, with whom she had a long standing and trusting relationship. Given the focus of this case study on collaborative practice, a detailed account of each collaboration is described below to provide information on the purpose of collaboration, what motivated it, and the decisions that ensued.

#### Collaboration 1 (May 2019)

The client’s first mental health care session with a CCT member occurred after she experienced a panic attack. Practitioners A (Support) and B (CCT Lead) were both out of the country at the time of this event thus the CCT agreed that Practitioner C (Support) would virtually meet with the client to perform a psychological assessment. Following this session, Practitioners A, B, and C held a videoconference to debrief the consult and collectively decide how to optimally support the client moving forward. Given that Practitioners A and B lived in the same city as the client, and the client was stable following the panic attack, the CCT decided that Practitioner B would remain the Lead and Practitioners A and C would remain Supports.

#### Collaboration 2 (August 2019)

The second instance of collaboration was triggered by an interpersonal conflict between the client and her peers in her training environment. This provoked the client to reach out to Practitioner A prior to her next scheduled appointment with Practitioner B. Practitioner A initiated a consultation with practitioner B to update her on the athlete’s experience. While the client expressed being more comfortable reaching out to Practitioner A given her previous mental performance work with her, both practitioners were committed to supporting the client and reviewing the client’s EHR to remain on the same page.

#### Collaboration 3 (September 2019)

The client completed four sessions with Practitioner B, after which she took a break. The client felt her symptoms had been resolved, so she discontinued all sessions for 3 months. On occasion, the client would informally check in with the MPC in the training environment, as the client had regular access to the MPC in this setting. The CCT discussed the client’s wishes and agreed that Practitioner A would let them know if and when the client was ready to resume care.

#### Collaboration 4 (February 2020)

The fourth collaborative event occurred after the client’s mood significantly declined, her anxiety spiked, and she expressed suicidal thoughts to Practitioner A. This prompted Practitioner A to suggest the client re-engage in a session with Practitioner C for clinical support. Practitioners A and C connected to update the client’s care plan, deciding that Practitioner A would continue regular mental performance sessions with the client in-person while practitioner C would perform additional clinical assessments *via* the virtual platform to better understand the client’s change in mood and onset of suicidal thoughts. While Practitioner B remained part of the CCT and had access to the client’s EHR, she did not have any more sessions with the client since the client felt there was a better fit with Practitioners A and C.

#### Collaboration 5 (February 2020)

A fifth collaboration was initiated by Practitioner A due to ongoing symptoms reported by the client. Practitioners A and C reflected on how to best support the client and decided that the client should meet with her family physician to discuss her medication. They agreed that Practitioner A would remain a local source of care given her established relationship and accessibility while Practitioner C would remain available as necessary.

#### Collaboration 6 (February 2020)

The sixth collaborative event occurred after the client disclosed suicidal thoughts once again to Practitioner A. Practitioners A and C met to follow up on the client’s progress and discussed how to keep her safe. Practitioner C recommended that the client see a local practitioner rather than continue to work with her remotely. Practitioners A and C met with the client together to explain the need for an external referral and obtained the client’s consent to share information with her physician so he could make a referral to a local psychologist and a psychiatrist. The practitioners ensured that their messaging was consistent and reassured the client that they would be there to support her throughout the transition.

#### Collaborations 7, 8, 9

There were additional instances of collaboration that shaped the client’s care until she transitioned out of the CCMHS. For example, Practitioners A and C conducted a few sessions together to help the client regulate her thoughts and mood. With the client’s consent, Practitioners A and C also had a session with the client’s parents to help guide them in supporting their daughter. The practitioners also worked together to craft emails to the client and her family. Lastly, Practitioner C consulted with a clinical psychologist specializing in suicidality and a psychiatrist to keep the client safe and make recommendations on medication management until she exited CCMHS care. Throughout their collaboration, Practitioners A and C kept each other informed of their approach and the client’s progress and they were also transparent about their collaborative work with the client.

## Practitioner reflections and discussion

Through practitioners’ reflective journals and the collaborative reflection exercise undertaken by the CCT, the processes, events, and outcomes surrounding collaboration in this case were revealed, as were factors that facilitated and impeded collaborative practice.

### Collaborative practice - processes, events, and outcomes

The CCT practitioners noted that the level of collaboration that occurred in this case fluctuated back and forth from independent provision of care (e.g., Practitioner B individually leading a session with the client), consultation/referral (e.g., Practitioner A consulting with Practitioner C and referring the client to her when her mood significantly declined in February 2020), to interdependent co-provision of care (e.g., Practitioners A and C co-leading sessions with the client). This parallels Jones and Way, (2006) structured collaborative practice model and shows how collaboration is a dynamic process that can change, even within one particular case. The three practitioners’ level of involvement varied throughout the 11-month period and was determined based on the client’s preferences and needs, as well as the occurrence of significant events. For example, practitioners observed that as the complexity and intensity of the client’s symptoms rose, increasingly close collaboration was also necessary:

*We needed to be in constant communication and share responsibilities in this case*. [Practitioner A] *being the practitioner in frequent and close contact with the client, providing instant and brief interventions when needed. And myself, acting as the clinical psychologist, building the client’s clinical comprehension, and stating the priority of interventions*. (Practitioner C)

Practitioners noted that their availability and scope of practice also influenced the collaboration that occurred throughout this case. For example, Practitioner B reflected on the adaptation that was required when the client’s mother reported an event in which the client was in distress and requested an earlier appointment: “Unfortunately, both Practitioner A and I happened to be out of the country for a few weeks [at that time]. Luckily, Practitioner C was available for an immediate telehealth session” (Practitioner B). In this instance the collaborative model allowed for the client to receive immediate care and facilitated continuity of care when she began regular sessions with Practitioner B. This also allowed Practitioners A and B to respect planned recovery activities knowing their client was cared for by a capable colleague, which contributed to these practitioners’ overall well-being and ability to provide quality care over time.

Additionally, the fluidity of the model allowed for the CCT to efficiently use their limited resources (e.g., time, emotional energy, client’s financial resources) to provide the best care possible and support one another to work within the boundaries of their professional competencies. As an MPC, Practitioner A highlighted the value of collaborating with the other members of the CCT:

*If I had been alone as an MPC on this case, I would have burnt out and felt nothing but anxiety, nervousness, worry, fixation, and self-doubt. I encountered so many novel challenges with this client that tested my ethics, boundaries of practice, personal assertiveness, and sense of competency (particularly when dealing with significant distress and suicidal ideation). Being able to reach out to a clinical psychologist colleague who was deeply involved with the same case, and thus, familiar with the client and concerns, allowed me to access support and reassurance that I was doing things correctly*.

As the previous citation suggests, support and recovery in the provision of mental health care are important (Dithurbide et al., 2022) and the CCMHS collaborative model affords opportunities to practitioners that may not exist when they work in isolation. The above quote also illustrates the demands that can be put on a single practitioner in the context of complex cases, which can lead to burnout if demands are not well managed (Maslach et al., 2001). Indeed, burnout is a threat to mental health service provision (Dreison et al., 2018), particularly when considered in the stressful context of the extended COVID-19 pandemic (Zunin and Myers, 2000). The CCMHS collaborative care model allows practitioners to lean on one another to not only develop the most effective care plans for clients but also support one another when well-deserved recovery periods are scheduled.

The collaborative care model and process yielded several positive outcomes for the client and CCT practitioners. For example, the model emboldened the client to initially seek mental health support because she was aware that Practitioner A could remain an integral member of her circle of care, and Practitioner A’s endorsement of the model and team enhanced the client’s trust in the referral process and ensuing care. Studies show that there are several barriers discouraging athletes to seek mental health care (Lopez and Levy, 2013; Delenardo and Lennox-Terrion, 2014). In this case, the MPC was instrumental in convincing the athlete to get clinical support through the CCMHS⎯ an important role that is also addressed in the Mental Health Strategy for High Performance Sport in Canada (Durand-Bush and Van Slingerland, 2021b) and in Dithurbide et al. (2022) study of Canadian national team athletes’ mental health and mental performance during the COVID-19 pandemic.

Collaboration also allowed the client to benefit from the team’s diverse and complementary skillsets, particularly as she experienced more distressing events at different time points: “Thanks to the willingness, flexibility and expertise of the Collaborative Care Team, the client was able to access a variety of services depending on her comfort, needs and level of psychological risk at the time” (Practitioner B). The collaborative model equally enabled the client to cognitively separate her work on mental performance from her work on mental health: “The client reported finding her work with Practitioner A helpful and wanting to continue using it as a place to focus on performance with our work directed towards managing [impairing] thoughts, emotions and interpersonal relationships” (Practitioner B). This is an important feature of the comprehensive CCMHS team that is comprised of a variety of mental performance and mental health specialists capable of maintaining focus on performance when the client’s state and context permit this. Of note, while all licensed mental health practitioners have foundational competencies to address a multitude of challenges and symptoms, practitioners typically do not deeply specialize in all mental disorder areas and treatments (e.g., eating disorders, trauma, bio-neurofeedback). The ease and timeliness with which additional practitioners with varying scopes of practice can be added to a client’s team *via* the CCMHS are certainly assets compared to practitioners who operate alone in private practice (Durand-Bush and Van Slingerland, 2021a). For instance, another noted outcome of the CCT’s integrated focus in this case was the onboarding of Practitioner C. She was able to quickly build a high-quality relationship with the client due to Practitioner A who shared the client’s history in advance of Practitioner C’s first meeting with the client: “[Practitioner A’s] feedback was really helpful for the establishment of a strong therapeutic alliance with the client. In fact, the client felt that we both cared about her” (Practitioner C).

Another important outcome of the collaborative model was that the CCT could regularly communicate and commiserate with one another as the athlete experienced significant events (e.g., suicidal thoughts) and the case became more complex and demanding: “It was so important for us to have one another to rely on, as the intensity of the client distress and the difficulty of the case would have been overwhelming alone” (Practitioner A). Practitioner C echoed this sentiment, reflecting in her journal that it was “helpful to realize that Practitioner A and I were facing the same obstacles in our work with the client.” Likewise, Practitioner B noted feeling confidence and relief knowing she had the support of other CCT members: “I knew I had peer consultation and collaboration available with other trusted practitioners who were also part of my client’s care team. That is a particular asset of the CCMHS model which I do not take for granted.” In her reflective journal, Practitioner A noted that without this collaborative process, the complexity of this case could have placed significant burden on a single practitioner working alone, which could have led to negative consequences for everyone involved. She attributed the provision of effective care in part to the opportunity to share the cognitive and emotional load of caring for this client with her colleagues. These observations regarding trust, empathy, support, and shared workload are congruent with that discussed by Van Slingerland and Durand-Bush (2021) in their study on the acceptability and appropriateness of the CCMHS model. The same attributes have been highlighted in the literature as benefits of collaborative mental health care (Markowski et al., 2021).

#### Factors facilitating collaboration

Practitioners identified several factors that facilitated a high degree of collaboration in this case, including (a) practitioner factors, (b) client factors, and (c) infrastructural factors. Practitioners perceived these factors to have enhanced the quality of client care provided.

##### Practitioner factors

Practitioner factors pertained to *individual practitioner characteristics* such as a willingness to collaborate and engage in professional reflection as well as *group characteristics* such as well-established professional relationships within the CCT and shared professional training as MPCs. Practitioners’ level of interest in and willingness to collaborate significantly contributed to the rate, intensity, and success of collaboration in this case. For example, Practitioner C noted that “[Practitioner A] was really available and she always took the time to answer really quickly.” This is particularly poignant because practitioners were not remunerated for the time they spent collaborating with one another, a drawback that was highlighted in the study conducted by Van Slingerland and Durand-Bush (2021). Similarly, individual competencies such as self-awareness and professional reflection aided in the collaborative process, as noted by Practitioner A:

*Being deeply aware of my own boundaries as an MPC helped me to accept where I had to stop work and had to allow [Practitioner C] in. But equally, having a good sense of where mental training could support mental health allowed [Practitioner C] and I to use our respective areas of expertise to support the client more regularly and consistently*.

The professional relationship established between the three CCT practitioners by virtue of working together through the CCMHS also facilitated collaboration because there was trust among them and familiarity with one another’s’ therapeutic style, skills, and strengths. This not only enhanced practitioners’ comfort with collaboration but also allowed them to direct the client to the team member best suited to help with particular challenges. This relationship was facilitated in part by infrastructural factors discussed below. Lastly, Practitioner A shared that the fact that all three CCT practitioners were trained as MPCs led to deeper collaboration because, “there was a certain level of understanding and appreciation for the work an MPC could do, which opened up more opportunity, sharing, and trust.” This illustrates the tremendous potential for MPCs and mental health practitioners to not only co-exist but also work together to optimally support athletes within Canada’s sport ecosystem—an observation equally made by Dithurbide et al. (2022) and Durand-Bush and Van Slingerland, 2021b. However, it is important to note that regardless of the collaborative care model used, teamwork should be founded on sound principles ensuring that “applicable provincial/territorial privacy, security, and confidentiality regulations are respected” (Durand-Bush and Van Slingerland, 2021b, p: 25).

##### Client factors

Client factors also facilitated collaboration among the CCT. For example, the client’s use of a single notebook to write her reflections and note key learnings, which she brought to care sessions with all providers, helped maintain continuity between practitioners: “The client has really good insight when she is in a good mood, which helps the interventions” (Practitioner C). Additionally, both practitioner A and B wrote about the client’s openness to collaborative care as facilitating the collaborative process: “The client’s openness to collaboration was facilitative to the CCT as well; she fully trusted us to engage in thorough collaboration and sharing, which allowed us to better support her needs in an efficient, non-redundant way” (Practitioner A). Clients’ willingness to work within a collaborative framework is essential. However, given the lack of integrated interdisciplinary mental health care models in sport, educating clients on the processes and potential outcomes of this type of care and allowing them to make an informed decision are central to ethical and successful practice. While several benefits of collaborative care have been highlighted thus far in this case, this format may not be the preferred choice of care for all athletes. As highlighted by Van Slingerland et al. (2020), clients referred to the CCMHS, including the current athlete forming this case, get briefed on the collaborative model used to deliver care and they are given a choice to pursue therapy or not with a team of practitioners. On the one hand, those who accept are asked to sign a consent form allowing information sharing between the practitioners on their CCT. On the other hand, those who decide not to continue with this type of care are referred to other mental health practitioners working independently within the sport community.

##### Infrastructural factors

Lastly, infrastructural factors that facilitated collaboration throughout this case included the CCMHS’ secure EHR system as well as policy and procedures surrounding team management and care provision. For example, the CCMHS’ virtual platform facilitated information continuity so that practitioners could deliver complementary interventions: “Practitioners were easily able to access each other’s notes through the Juno EMR online system. This enabled more in-sync and relevant practice” (Practitioner B). Embedded processes and procedures also aided collaboration. For instance, regular team meetings with all CCMHS practitioners that included case presentations engendered familiarity and trust between CCT members. Further, information sharing policies and procedures including internal and external consent forms ensured ethical and timely collaboration within the client’s CCT and between the CCT and other experts. As an example, when Practitioner C took a leading role in the client’s care after a series of significant events, Practitioner A was able to provide the client’s history and relay core concerns as well as the client’s preferred therapeutic approach, allowing Practitioner C to begin her relationship from an informed perspective: “Practitioner A’s insights about the client allowed me to adapt my interactions with her. It was also really helpful for building my clinical comprehension of the client’s difficulties.” Similarly, when the intensity of the client’s symptoms reached concerning thresholds, third-party information sharing procedures and the consent provided by the client during intake allowed Practitioner A to consult with a member of the larger CCMHS team with expertise in suicidal ideation. It also enabled Practitioner C to consult in a timely manner with a sport psychiatrist external to the CCMHS team and the client’s family physician to discuss the client’s medication and recommend referral to psychiatry. These favourable infrastructural factors appear to be important as they were highlighted in another case study with a different CCMHS client (an athlete) and collaborative care team comprised of both mental performance and mental health practitioners (Van Slingerland et al., 2020). Similar factors were also noted in the Mental Health Strategy for High Performance Sport in Canada, wherein a key action recommended by a group of mental health and sport experts was to “create policies and procedures including but not limited to billing, invoicing, information sharing and inter-provincial case management, data tracking, communication with clients, care provision (e.g., consents/assents), and virtual care delivery (Durand-Bush and Van Slingerland, 2021b, p: 25). As such, attention should be paid to optimize infrastructure in collaborative care environments.

#### Factors impeding collaboration

Practitioners also identified factors that impeded collaborative processes in this case, which also pertained to client and infrastructural factors. These factors were perceived to challenge the CCT’s efficacy when providing care at certain times during the therapeutic process.

##### Client factors

With regards to client factors, there were certain characteristics including the client’s challenges with building trusting relationships, her age, and her acute symptomology at times that challenged practitioners’ ability to collaborate effectively. For example, the client had formed a strong relationship with her MPC (Practitioner A), causing her to rely on Practitioner A in times of great distress. This was not only emotionally difficult for Practitioner A, but also hindered the client’s improvement. Practitioner A noted, “I had to set an imperative boundary with the client so she would stop reaching out to me in distress and start taking initiative to book her sessions with Practitioner C and actually engage with the therapeutic work we were trying to do.” The client’s age also added a layer of complexity as her relative youth (chronologically and developmentally) often necessitated inclusion of and communication with a parent (her mother) to ensure her safety and continued progress towards therapeutic goals. Lastly, the nature of the symptoms the client was experiencing challenged her own and practitioners’ efficacy to achieve therapeutic process. Practitioner C shared, “It is hard to help the client when she is in a bad mood, because she easily feels misunderstood or invalidated in how she feels. Her main coping mechanisms are emotional avoidance and withdrawal from others.” These client characteristics highlight the complexity of providing care to youth athletes. It appears that the potential to develop dependency should remain at the forefront of collaborative care teams so that challenges can be addressed as early as possible. Furthermore, the implication of a parent was deemed critical in this case when safety was an issue. This is in line with national recommendations, which emphasize that involving family in therapy, providing education on suicidality and co-occurring mental health symptoms or illness, and providing support and resources for family members are considered best practices for individuals with suicide risk (National Action Alliance for Suicide Prevention, n.d.). Within the sport context, more research is required to examine collaboration with athletes’ extended support network such as parents and coaches as studies shedding light on their involvement and impact on mental health outcomes are sorely lacking.

##### Infrastructural factors

In terms of infrastructure, practitioners’ reflections revealed that the CCMHS’ remuneration model and the geographical distance between the client and one member of the CCT, and between the three practitioners impaired collaboration in certain instances. For example, the client’s psychological risk paired with her inability to meet with the psychologist in person placed additional strain on Practitioner A who was the only provider that could offer immediate in-person support (outside of emergency services): “The fact that the client eventually felt that she could not open up to Practitioner B because she [the client] had “halted” that relationship made it difficult, because I had to become the ‘on the ground’ practitioner and only collaborate with [Practitioner C]” (Practitioner A). Similarly, practitioners’ physical distance from one another was perceived to limit the full range of advantages of collaborations that would be possible if they worked together in person: “I can imagine if practitioners happened to be working physically under one roof together, there would be additional opportunities to build our professional relationships with each other and maybe even further collaborate on cases such as our work with the client” (Practitioner B). This demonstrates that although significant strides have been made to offer high-quality virtual mental health care (Langarizadeh et al., 2017), particularly as a result of the COVID-19 pandemic (Dithurbide et al., 2022), in-person interactions remain important and necessary in times of distress (e.g., recurring suicidality; Madigan et al., 2020; National Action Alliance for Suicide Prevention, n.d.). Future research should examine the necessity and impact of using different modalities to collaborate and provide services to athletes who are often travelling and may only be able to communicate through an online platform. Finally, practitioners also recognized that despite many benefits of collaboration, it was unfortunate that the CCMHS model could not allow them to be compensated for any work that did not involve directly interacting with the client. The lack of remuneration for the collaborative aspect of mental health care remains a challenge not only for the CCMHS⎯ a charity that does not receive any government funding⎯ but also for other interdisciplinary primary care teams in Canada (Wranik et al., 2017).

## Conclusion

This case study offers valuable knowledge and practical implications for practitioners and leaders seeking to increase their understanding of collaborative mental health care and to develop novel interdisciplinary approaches to improve mental health outcomes in sport. The details provided to illustrate and interpret the current case shed light on the variability, complexity, and feasibility of collaboration between mental performance and mental health practitioners. The CCMHS’ model to foster integrated interdisciplinary work amongst its practitioners is unique in Canada and the world. Benefits of collaboration reported by the current care team included (a) autonomy and flexibility in the type and extent of collaboration that occurred, (b) trustworthy relationships, various skillsets, and complementary scopes of practice between practitioners, and (c) ongoing support, understanding, and shared workload, particularly when facing challenges during the 11-month service provision timeframe. Factors facilitating collaboration pertained to client and practitioner characteristics (e.g., willingness to engage in collaborative care and self-reflection) and infrastructural characteristics (e.g., secure EHR system and sound policies and procedures). Interestingly, other client characteristics such as over-reliance on a practitioner, age, and complex symptomology challenged the team’s collaboration. Furthermore, geographical distance between members of the team and the client impeded progress at times. Lastly, appropriate compensation for the high-quality collaborative work performed by this team was recommended to incite additional teamwork and the adoption of more collaborative frameworks in sport. Key themes that should be further explored in the future to optimize collaborative mental health care in sport are autonomy, complementarity, financial and emotional support, and delivery modality. The care coordinator role also deserves empirical attention given that the person in this position appears to serve as the ‘glue that binds everything together’. Finally, contextual factors must be further studied, particularly since many efforts are being deployed worldwide to improve mental health outcomes in sport. According to Larsen et al. (2021), there is considerable variability across countries when it comes to mental health and this is influenced by factors such as resources, culture, leadership, and competencies, to name a few. It is our hope that this case study will inspire individuals to advocate for the development of collaborative frameworks that enable mental performance and mental health practitioners to work with clients in a safe and synergic fashion to not only provide high quality care but also to reduce inefficiencies and common ‘turf wars’ within sport systems.

## Data availability statement

The datasets presented in this article are not readily available in order to protect participant anonymity and privacy. Requests to access the dataset should be directed to ndbush@uottawa.ca.

## Ethics statement

The studies involving human participants were reviewed and approved by the University of Ottawa Research Ethics Board. Written informed consent to participate in this study was provided by the participant.

## Author contributions

All authors collaborated to conceptualize the overall idea and study design. KV, PD, VB, and AA collaborated on data collection while KV led data analysis, interpretation, and integration. ND-B and PD also contributed to data analysis, interpretation, and integration, acting as “critical friends,” and to the writing of the manuscript by verifying client data, ensuring anonymity was respected, and connecting study findings with the literature. All authors contributed to the article and approved the submitted version.

## Conflict of interest

The authors declare that the research was conducted in the absence of any commercial or financial relationships that could be construed as a potential conflict of interest.

## Publisher’s note

All claims expressed in this article are solely those of the authors and do not necessarily represent those of their affiliated organizations, or those of the publisher, the editors and the reviewers. Any product that may be evaluated in this article, or claim that may be made by its manufacturer, is not guaranteed or endorsed by the publisher.

## Footnotes

^1^Mental health is a state of psychological, emotional, and social well-being in which individuals are capable to feel, think, and act in ways that allow them to enjoy life, realize their potential, cope with the normal stresses of life, work productively, and contribute to their community (Public Health Agency of Canada, 2006).

^2^Mental performance refers to the capability with which individuals use cognitive processes (e.g., decision-making, perception, reasoning) and mental/self-regulation competencies (e.g., goal-setting, self-confidence, communication) to perform in their changing environment (Durand-Bush and Van Slingerland, 2021b).

^3^Mental illness is a health condition characterized by alterations in thinking, mood, and/or behaviour associated with significant distress and impaired functioning. It collectively refers to all diagnosable mental disorders (e.g., depression, anxiety disorders; Public Health Agency of Canada, 2006).

^4^Additional information regarding the athlete and her sport are not provided so as to not potentially violate anonymity.
